# Minimum acceptable diet among children aged 6–23 months in South Kivu, Democratic Republic of Congo: a community-based cross-sectional study

**DOI:** 10.1186/s12887-021-02713-0

**Published:** 2021-05-19

**Authors:** Richard Mbusa Kambale, Gaylord Amani Ngaboyeka, Joe Bwija Kasengi, Sarah Niyitegeka, Boss Rutakaza Cinkenye, Armand Baruti, Kizito Chentwali Mutuga, Dimitri Van der Linden

**Affiliations:** 1 Institute of Experimental and Clinical Research, Université Catholique de Louvain, Brussels, Belgium; 2 Université Catholique de Bukavu, Bukavu, Democratic Republic of Congo; 3 Pediatric Department, Hôpital Provincial Général de Référence de Bukavu, Bukavu, Democratic Republic of Congo; 4 Pediatric Infectious Diseases, General Pediatrics, Pediatric Department, Cliniques universitaires Saint Luc, Brussels, Belgium

**Keywords:** Complementary feeding, Infants, Minimum acceptable diet, Nutrition, South Kivu

## Abstract

**Background:**

Suboptimal child nutrition remains the main factor underlying child undernutrition in Democratic Republic of Congo (DRC). This study aimed to assess the prevalence of minimum acceptable diet and associated factors among children aged 6–23 months old.

**Methods:**

Community-based cross-sectional study including 742 mothers with children aged 6–23 months old was conducted in 2 Health Zones of South Kivu, Eastern DRC. WHO indicators of Infant and Young Child Feeding (IYCF) regarding complementary feeding practices were used. Logistic regression analysis was used to quantify the association between sociodemographic indicators and adequate minimum acceptable diet for both univariate and multivariate analysis.

**Results:**

Overall, 33% of infants had minimum acceptable diet. After controlling for a wide range of covariates, residence urban area (AOR 2.39; 95% CI 1.43, 3.85), attendance postnatal care (AOR 1.68; 95% CI 1.12, 2.97), education status of mother (AOR 1.83; 95% CI 1.20, 2.77) and household socioeconomic status (AOR 1.72; 95% CI 1.14, 2.59) were factors positively associated with minimum acceptable diet.

**Conclusion:**

Actions targeting these factors are expected to improve infant feeding practices in South Kivu.

**Supplementary Information:**

The online version contains supplementary material available at 10.1186/s12887-021-02713-0.

## Introduction

Sustainable Development Goals (SDG) 2 and 3 call for an ending all forms of undernutrition, and all preventable deaths under 5 years of age by 2030 [1]. Worldwide, 5.4 million children under-five still die each year, 80% of them in sub-Saharan Africa and South Asia. Almost half of these deaths occur among undernourished children [2, 3]. Although these deaths are the result of a complex set of determinants, poor breastfeeding practices and inadequate complementary feeding play a major role [4]. Therefore, optimal infant and young child feeding (IYCF) practices rank among the most effective interventions to improve child health.

In 2002, the World Health Organization (WHO) and United Nations International Children’s Emergency Fund (UNICEF) adopted the global strategy for IYCF, including: (i) early initiation of breastfeeding within 1 h of birth; (ii) exclusive breastfeeding for the first 6 months of life and (iii) introduction of nutritionally-adequate and safe complementary (solid) foods at 6 months together with continued breastfeeding up to 2 years of age or beyond [5].

The Democratic Republic of Congo (DRC) is still recovering from years of war and political upheaval and continues to face significant humanitarian challenges. Since 2016, the long-running crisis in Eastern regions has forced some 4.8 million internally displaced persons to flee from their villages and lose their agricultural livelihoods and jobs. The number of food-insecure people almost doubled from 7.7 million in 2017 to 13.1 million in 2018, making access to food a daily struggle for a significant part of the Congolese population [6]. The health and nutritional status of the children under 5 years of age are have been impacted by these years of armed conflict and instability: approximately 42% are stunted, 6% are wasted, 23% are underweight, and 47% are anemic (< 11.0 g/dl); Among children 6–23 months, only 18 and 37% receive the WHO recommended minimum dietary diversity and meal frequency each day, respectively; Furthermore, the under-5 mortality rate is 70 per 1000 live births [7]. Thus, undernutrition and suboptimal IYCF practices remain serious public health concerns in DRC.

To address inadequate IYCF practices, the Ministry of Public Health has developed a program named “Infant and Young Child Feeding in Emergency”. This program aimed to provide concise, practical guidance on how to ensure appropriate IYCF in emergencies [8].

To date, there has been no published report on infant complementary feeding practices in South Kivu, a city that has the highest stunting rate among children under 5 years of age in DRC (48%) [7]. Yet growing evidence suggests that suboptimal IYCF practices may be a major contributor to the high prevalence of stunting in Eastern DRC [9–11]. Therefore, context-specific information on infant complementary feeding practices is needed as eating habits differ according to the tribes and customs in DRC. This study sought to assess the prevalence of minimum acceptable diet, as well as identify associated factors of adequate minimum acceptable diet, among children under 2 years of age in two health zones in South Kivu, Eastern DRC. Apart from a better understanding of the realities of infant complementary feeding practices, this study could generate evidence of a better practice of IYCF in an economic, political, social, and health context of precariousness, particularly to the context of the DRC.

## Methods

### Study design and setting

This community-based cross-sectional study was conducted in August 2019 in 2 Health Zones of South Kivu (Eastern DRC): Ibanda Health Zone, an urban area, and Kabare Health Zone, a rural one. The Health Zone is a geographical area contained within the limits of an administrative territory, comprising a population of at least 100,000 inhabitants. A Health Area is a geographical area consisting of a group of villages (in rural areas) or streets (in urban areas) with a population size of 10,000 inhabitants [12]. Ibanda Health Zone was selected by simple random sampling from the 3 Health Zones of Bukavu urban city (Kadutu, Bagira, Ibanda); Kabare Health Zone was selected also by simple random sampling from 5 surrounding rural areas of Bukavu (Nyantende, Walungu, Kabare, Katana, Miti-Murhesa). Each of these 2 selected Health Zones encompasses 16 Health Areas.

The Ibanda Health Zone is located in the municipality of Ibanda, city of Bukavu, in the South Kivu province of DRC. Bounded on the north by Lake Kivu and on the east by Rwanda, Ibanda Health Zone is an area with mountainous terrain, clay soil, grassy vegetation and a tropical highland climate. At the last census in 2018, it had a recorded population of 452,608 with a population density of 32 per km^2^. Infants < 24 months of age were estimated at 10,275. The main activities are small-scale trading and administration. Secondary activities are subsistence agriculture, small livestock farming and artisanal fishing. The staple food is cassava, cereals (maize, rice and sorghum), other tubers (taro, sweet and white potatoes) and bananas. These foods are generally served with vegetables, fish, beans and meat, which helps to balance the family dish. On average, two to three meals are consumed per day [13].

Kabare Health Zone is located in the Kabare administrative zone of South Kivu province in the DRC. It is situated 17 km from the city of Bukavu. It is an area with high plains and hills at elevations between 900 and 1900 m, with a tropical highland climate. At the last census in 2018, it had a recorded population of 213,882 with a very high population density of 856 per km^2^. Infants under 24 months of age were estimated at 12,800. The population of this area is devoted to agriculture, livestock and small-scale trade. Bananas, cassava, taro, sweet and white potatoes, corn and sugar cane are the main crops grown there. However, the soil is not very fertile, and most of the population of the area leaves the fields to sell labor in the city of Bukavu [14].

### Eligibility criteria

The study included mothers with children aged 6–23 months old with the following characteristics: (i) Infants aged between 6 and 23 months; (ii) residence in the study area; (iii) infants without chronic debilitating illnesses such as cerebral palsy, congenital heart disease, Down syndrome, cleft lip/palate; and (iv) parental consent.

### Sample size

The sample size was calculated using Emergency Nutrition Assessment 2011 software, considering an absolute precision of 5% at the 95% confidence level, a design effect of 1.5 and a 52% prevalence of minimum acceptable diet, estimated based on a study conducted in Western Indonesia [15]. Allowing for 20% refusals and incomplete questionnaires, the required sample size was 750. Thus, we projected a sample of 375 in each Health Zone.

### Sampling procedure of the study

In each Health Zone, a complete list of households with an infant between 6 and 24 months of age was obtained through a prior survey. This list served to identify and create sampling frame and apply the systematic random sampling to select the study samples, in order to assure the selection of representative sample from each health area.

Ten health extension workers and one public health professional were recruited as data collector and supervisor respectively. Data were collected in a separate room, in the absence of other relatives. We used face-to-face interview during house-to-house visit from mothers who had an infant between 6 and 24 months of age using structured questionnaire. The questionnaire contained the information on sociodemographic characteristics of participants and infant feeding practices as proposed by WHO. To determine household socioeconomic status, a household wealth score was computed as proposed by Bangirana et al. [16] based on the material possession of several items (electricity, radio, bike, motorbike, car) and on several characteristics of the house such as material used for the walls and for the roof. The wealth score of a household was obtained by summing points attributed to the above items and ranged from 1 to 11. We determined the child’s age based on the date of birth (obtained either from birth certificate, child health record booklet or baptismal card) and the date of the survey. The mother’s school level was subdivided into two classes: low (for mothers unschooled or primary school), and good (for those having secondary school or university). The household socioeconomic status was categorized into two classes: good (for households with medium or high socioeconomic level), and low.

For data quality control, the questionnaire was first developed in French and translated to local language (Swahili and Mashi language) and then back-translated to French by an independent translator for consistency. French version of the survey is provided in the Supplementary Appendix. Training was given to health extension workers and supervisor for 2 days. The questionnaire was pre-tested in 19 (5%) mothers in Ibanda Health Zone and 19 (5%) mothers in Kabare Health Zone, which were not included in actual study, to assess the content and approach of the questionnaire. To assure the quality of the data, the supervisor and investigator closely reviewed the data collection technique on daily basis, reviewed the filled questionnaire for completeness and returned any incomplete questionnaire to the data collectors for correction. There was also debriefing every day.

### Operational definitions

#### Introduction of solid, semi-solid or soft foods

Children 6–7 months who received solid, semi-solid or soft foods at least once on the day preceding the survey date [17].

#### Minimum meal frequency

Breastfed children 6–23 months who were fed a minimum recommended number of times in the previous day of the survey. Minimum is defined as 2 times for breastfed infants 6–8 months, 3 times for breastfed children 9–23 months, 4 times for non-breastfed children 6–23 months [17].

#### Minimum dietary diversity

Children 6–23 months who received in the previous day of the survey four or more food groups out of seven food groups: (i) grains, roots and tubers; (ii) legumes and nuts; (iii) dairy products (milk, yogurt, cheese); (iv) eggs; (v) flesh foods (meat, fish, poultry and liver/organ meats); (vi) vitamin-A-rich fruits and vegetables; and (vii) other fruits and vegetables [17]. WHO considers the cut-off of at least 4 of the above 7 food groups above because the consumption of foods from at least 4 food groups would mean that in most populations the child had a high likelihood of consuming at least one animal-source food and at least one fruit or vegetable that day, in addition to a staple food (grain, root or tuber) [17].

#### Minimum acceptable diet

Children 6–23 months old who met age-specific minimum recommended diet diversity and minimum recommended meal frequency and consumed a source of dairy (or were breastfed) in the previous day of the survey [17]. The minimum acceptable diet indicator was expressed as dichotomous variable categorized as “adequate minimum acceptable diet” and “inadequate minimum acceptable diet”. A child who met both the minimum dietary diversity and the minimum meal frequency was categorized as “adequate minimum acceptable diet”, and a child who did not meet either or both the minimum dietary diversity and the minimum meal frequency as “inadequate minimum acceptable diet”.

### Statistical data analyses

Data were entered and analyzed using Statistical Package for the Social Sciences for Windows version 25 (SPSS Inc. Version 25.0, Chicago, Illinois). Characteristics of mothers and infants were summarized as mean and standard deviation (SD) for continuous variables with a normal distribution, or as median and range for continuous variables with a non-normal distribution, and as number or percentages for categorical variables. Normality of continuous variables was explored visually (Q-Q plots and histogram) and numerically (Shapiro-Wilk and Kolmogorov-Smirnov tests). Proportions were compared by using either the χ^2^ or the Fisher exact test. The sociodemographic variables were selected on the basis of previous literature. They were imported into the multiple regression model on the basis of a value *p* ≤ 0.25 and/or on the basis of biological plausibility. The unadjusted and adjusted odds ratios with their 95% confidence intervals were used to measure the association between the variables and minimum acceptable diet. The significance level was fixed at 0.05.

### Ethical considerations

This study was conducted according to the guidelines laid down in the Declaration of Helsinki and all procedures involving research study participants were approved by the Ethical Committee of the Université Catholique de Bukavu (UCB/CIES/NC/08/2018). Informed consent was obtained from all study participants as well as from infants’ parents.

## Results

The study included 742 mothers with children aged 6–23 months old (371 in Kabare Health Zone and 371 in Ibanda Health Zone), yielding a 98.9% response rate. Their sociodemographic characteristics are summarized in Table 1.

**Table 1:** Sociodemographic characteristics of the study participants

Characteristics	Total (*N* = 742)
Age (in months), mean (Standard Deviation)	16 (7.8)
6–12 months, n (%)	252 (34.0)
13–23 months, n (%)	490 (66.0)
Sex of the child	
Male, n (%)	381 (51.3)
Female, n (%)	361 (48.7)
The child lives with	
Both biological parents, n (%)	691 (93.1)
The mother only, n (%)	51 (6.9%)
Age of mothers age (in months), median (range)	26 (17–41)
Interval between last and before last births (months), mean (SD)	29 (17)
Place of birth	
Hospital, n (%)	224 (30.2)
Health Center, n (%)	467 (62.9)
Home delivery, n (%)	51 (6.9)
Attendance at postnatal visits	
Yes, n (%)	631 (85.0)
No, n (%)	111 (15.0)
Household socioeconomic status	
High, n (%)	15 (2.0)
Medium, n (%)	133 (18.0)
Low, n (%)	594 (80.0)
Mother educational status	
Maternal education more than secondary, n (%)	450 (60.6)
Maternal education less than secondary, n (%)	292 (39.4)
Polygamy	
No, n (%)	466 (62.8)
Yes, n (%)	276 (37.2)
Number of household members, median (range)	6 (4–14)
Sibling size, mean (Standard Deviation)	5 (3)

Eighty percent of households had a low socioeconomic level, and 55.8% of mothers had a low educational level. Low socio-economic and maternal educational levels were more prevalent in rural areas than in urban ones (*p* < 0.05). The postnatal care follow-up rate was 85.0%, higher in rural areas than in urban ones (*p* < 0.05).

**Fig. 1:**
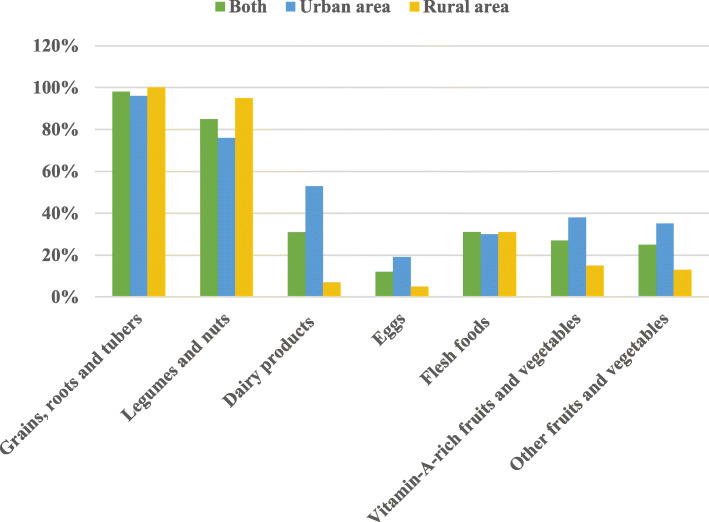
Proportion of children consuming the different food groups in South Kivu (DRC), 2019; *N* = 742

Complementary feeding practices are summarized in Fig. 1 and Table 2. The majority, 727 (98%) and 631 (85%) infants received cereals-roots-tubers and legume-based foods respectively. The consumption of animal foods was low as only 31% consumed flesh foods and only 12% consumed eggs. Consumption of dairy products, eggs, fruits and vegetables was significantly lower for rural infants compared to urban ones (*p* < 0.05). About 64% of infants had minimum meal frequency, 34% had minimum dietary diversity, and 33% had minimum acceptable diet, with rates higher in urban area than in rural one for all these indicators (*p* < 0.05).

**Table 2:** Proportions of children meeting the WHO complementary feeding indicators in South Kivu (DRC), 2019, *N* = 742

Variable	Total n (%)	Urban n (%)	Rural n (%)
Introduced solid, semi-solid or soft foods (children 6–7 months)	76/100 (76.0)	41/53 (77.3)	35/47 (74.5)
Took minimum meal frequency (children 6–23 months) **	474/742 (63.9)	294/397 (74.0)	180/345 (52.2)
Took minimum dietary diversity (children 6–23 months) **	250/742 (33.7)	179/397 (45.0)	71/345 (20.6)
Took minimum acceptable diet (children 6–23 months) **	245/742 (33.0)	155/397 (39.0)	90/345 (26.0)
Consumed iron-rich foods (children 6–23 months) **	372/742 (50.1)	227/397 (57.2)	145/345 (42.0)

*: *p* < 0.05; **: *p* < 0.01

The multivariate analysis showed that residence area, attendance at postnatal care, household socioeconomic status, and maternal education status were statistically associated with minimum acceptable diet.

Mothers living in urban area were 2.36 times [Adjusted Odds Ratio (AOR) 2.39; 95% CI 1.43, 3.85] more likely to provide minimum acceptable diet to their child when compared to their counterparts. Similarly, higher odds of receiving minimum acceptable diet was observed among mothers who had postnatal care visits (AOR 1.68; 95% CI 1.12, 2.97). Likewise, mothers with secondary and post-secondary education were 1.83 times (AOR 1.83; 95% CI 1.20, 2.77) more likely to give recommended minimum acceptable diet compared to their counterparts. Finally, higher odds of receiving minimum acceptable diet was observed among mothers with good household socioeconomic status (AOR 1.72; 95% CI 1.14, 2.59) (Table 3).

**Table 3:** Factors associated with no minimum acceptable diet in South Kivu (DRC), 2019, *N* = 742

Variables and categories	Minimum acceptable diet		COR (95%CI)	AOR (95%CI)
	Inadequate, n (%)	Adequate, n (%)		
**Residence Health Zone**				
Rural	289 (83.5)	56 (16.5)	1	1
Urban	186 (53.5)	185 (46.5)	5.13 (3.18–6.29) *	2.39 (1.43–3.85) *
**Place of delivery**				
Home	29 (58.8)	21 (41.2)	1	1
Health facility	472 (62.2)	220 (31.8)	0.64 (0.21–1.34)	
**Attended PNC**				
No	92 (82.9)	19 (17.1)	1	1
Yes	409 (64.9)	222 (35.1)	2.63 (1.25–4.89) *	1.68 (1.12–2.97) *
**Socioeconomic status**				
Low	434 (72.9)	160 (27.1)	1	1
Good	64 (44.8)	79 (55.2)	3.35 (2.28–4.88) *	1.83 (1.20–2.77) *
**Maternal education**				
Less than secondary	242 (82.9)	50 (17.1)	1	1
Secondary and above	258 (57.5)	192 (42.5)	3.60 (2.50–5.49) *	1.72 (1.14–2.59) *
**Polygamous family**				
Yes	226 (81.9)	50 (18.1)	1	1
No	275 (59.0)	191 (41.0)	3.14 (2.24–4.67) *	
**Parents live with children**				
No	33 (67.3)	16 (32.7)	1	1
Yes	468 (67.6)	225 (32.4)	0.99 (0.81–1.27)	
**Household size**				
> 10	77 (72.0)	30 (28.0)	1	1
5–10	238 (66.2)	122 (33.8)	1.24 (0.76–2.03)	
< 5	186 (67.4)	89 (32.6)	0.96 (0.69–1.34)	

*AOR* Adjusted Odds Ratio, *CI* Confidence interval, *COR* Crude Odds Ratio, *PNC* Postnatal care

*p < 0.05

## Discussion

This study sought to assess the prevalence and associated factors of adequate minimum acceptable diet among children 6–23 months. Findings revealed that the prevalence of minimum acceptable diet was 33%. Residence in urban area, good maternal education status, good household socioeconomic status, and attendance to postnatal consultations were factors that may increase the minimum acceptable diet practices.

The prevalence of minimum acceptable diet found in this study is close to that reported in Nepal (29%) [18] and China (27%) [19]. Minimum acceptable diet prevalence ranging from 8 to 18% have been observed in India, Bhutan, Ethiopia and Malawi [20–23], while in Tanzania [24], Bangladesh [25], and Indonesia [15] they were 38, 42, and 48% respectively. The differences in health services, including health education and advice on breastfeeding and complementary feeding during prenatal and postnatal care, and region-dependent feeding cultural practices could be the factors explaining these differences.

The acute nature of emergency situations such as those observed in Eastern DRC challenges optimal feeding practices. They may increase the likelihood of not breastfeeding, as well as the risks of artificial feeding and inappropriate complementary feeding, with potentially devastating short- and long-term implications on malnutrition, illness and mortality [26].

In our report, urban area appeared better in several aspects of complementary feeding practices than rural area. This might be the result of cumulative effect of a series of more favorable conditions, including better socioeconomic and educational conditions, in turn leading to better caring practices for children and their mothers. A study analyzing Demographic and Health Surveys (DHS) from 36 developing countries revealed that for the same reasons, the prevalence of malnutrition was higher in rural than in urban areas [27].

The association between attendance at postnatal care and adequate minimum acceptable diet is consistent with study findings in East African region and India [28–32]. Mothers’ education on complementary feeding practices during these visits is the factor underlying this association. In DRC’s health system, children’s growth monitoring is conducted in every health facility according to the national nutrition program. In the outreach clinics, health workers monitor the weight of children using growth charts, and provide nutrition education to mothers or caregivers of children. These activities also provide an opportunity to discourage harmful traditional beliefs that might inhibit child feeding practices, and to early recognition of signs of undernutrition, any illness and manage them accordingly [33].

Our findings show that more than 85% of the mothers attended at postnatal consultations. Surprisingly, the prevalence of minimum acceptable diet remained low. The low household socioeconomic status could be the main explanatory factor. Indeed, our study showed that about 80% of households had a low socio-economic status. This finding emphasizes the role of socio-economic status on feeding practices. Households with high socioeconomic status are more likely to be food secure, thus they can afford to provide the minimum acceptable diet to their children [34]. Several studies have also shown that improved household wealth has a significant effect on adequate complementary feeding practices [31, 35–38].

Consistent with studies conducted in China [19] and Ethiopia [39], the current study also found that mothers with secondary and post-secondary education had higher odds of adequate minimum acceptable diet. The possible reason might be that education is directly linked to women’s autonomy, changes in traditional beliefs and women’s control over household resources [40]. Hence educated mothers are more likely to understand information provided through health and nutritional programmes regarding complementary feeding practices [41].

Three systematic reviews [42–44] examined the effectiveness of infant’s complementary feeding interventions. The socioeconomic status of caregivers, maternal education level, and postnatal care have been identified as main factors that may influence infant’s complementary feeding practices. Nevertheless, the results of the 3 systematic reviews demonstrated that interventions that provide education to mothers will significantly improve complementary feeding knowledge and practices. When population is food secure, educational strategies alone can improve feeding practices. The case is different in the context of food insecurity or political trouble.

### Study limitations

Our study has several limitations. Firstly, the recall bias may be possible and affect the validity of the results. However, this bias was minimized since most data related to infant’s complementary feeding practices were based on a 24-h recall method. Another limitation is the social desirability bias. Finally, the cross-sectional design does not allow for causal inference.

## Conclusion

In the context of South Kivu (DRC), our findings reveal a low prevalence of adequate minimum acceptable diet among infants between 6 and 23 months of age. Overall, the residence area, mother’s educational status, household wealth, and postnatal care attendance were factors positively associated with adequate minimum acceptable diet. These findings suggest policy-makers to focus on improving and making effective behavior change, strengthening education of mothers, as well as improving complementary feeding practices for children through food security, health care utilization, and improved household socioeconomic status.

## Supplementary Information

**Additional file 1.**

## Data Availability

The datasets used and/or analyzed during the current study are available from the corresponding author on reasonable request.

## Abbreviations

AOR: Adjusted odds ratio
CI: Confidence interval
DRC: Democratic Republic of Congo
IYCF: Infant and young child feeding
MICS: Multiple indicator cluster survey
OR: Odds ratio
PNC: Postnatal care
SPSS: Statistical package for the social sciences
WHO: World Health Organization
UNICEF: United Nations Children’s Fund
