# Supplementary material for: Minimum acceptable diet among children aged 6–23 months in South Kivu, Democratic Republic of Congo: a community-based cross-sectional study
Source: BMC Pediatr. 2021 May 19;21:239. doi: 10.1186/s12887-021-02713-0 (PMC8132412; doi:10.1186/s12887-021-02713-0)
Supplement: Supplementary file 1 [file 12887_2021_2713_MOESM1_ESM.doc]

FICHE D'ENQUETE: ALIMENTATION DU NOURRISSON ET DU JEUNE ENFANT (ANJE)

ID de l'enfant ______________________ Zone de santé ______________________ Aire de santé ______________________

Age (en mois) [ ] Sexe [ ] 1=M; 2=F Age de la mère (ans) [ ] Taille de ménage [ ] Taille de la fratrie [ ]

L'enfant vit avec qui? [ ] 1= Les 2 parents; 2= père; 3 = mère; 4= grand-mère; 5= autre Statut marital [ ] 1= Marié; 2= célibataire; 3= divorcé; 4=veuf(ve); 5= vivent en couple

Type mariage [ ] 1= monogamie; 2 = polygamie Lieu d'accouchement [ ] 1= Hôpital; 2 = Centre de Santé; 3= A la maison Intervalle inter genesique (mois) [ ]

La mère a-t-elle suivi les consultations postnatales? [ ] 1= Oui; 2=Non Niveau d'étude de la mère [ ] 1= analphabète; 2= primaire; 3= secondaire; 4= Universitaire

Présence d'électricité à la maison [ ] 1= Oui; 2=Non Radio [ ] 1= Oui; 2=Non Vélo [ ] 1= Oui; 2=Non Moto [ ] 1= Oui; 2=Non Voiture [ ] 1= Oui; 2=Non

Matériau principal du plancher: [ ] 1=Revêtement naturel (terre, sable, fumier); 2=Revêtement rudimentaire (planches de bois); 3=Revêtement de sol (ciment, tapis, parquet ou bois poli)

Matériau principal de la toiture [ ] 1=Toiture naturelle (sans toit, chaume, feuille de palmier); 2= Toiture rudimentaire (natte rustique, palmier, bambou, planche de bois); 3= Toiture finie (métal, bois, ciment)

Matériau principal des murs extérieurs [ ] 1= Murs naturels (canne, palmier, troncs); 2= Murs rudimentaires (bambou avec boue, pierres avec boue); 3= Murs finis (ciment, pierres avec chaux/ciment, briques)

Sous allaitement maternel [ ] 1= Oui; 2=Non Nombre des repas consommés la veille [ ] Age d'introduction d'aliments complémentaires [ ]

Aliments consommés au petit déjeuner ....

Aliments consommé à midi ....

Aliments consommé le soir ....

Alimentse consommés lors de la collation ....
